# Binary Silicone Elastomeric Systems with Stepwise Crosslinking as a Tool for Tuning Electromechanical Behavior

**DOI:** 10.3390/polym14010211

**Published:** 2022-01-05

**Authors:** Adrian Bele, Liyun Yu, Mihaela Dascalu, Daniel Timpu, Liviu Sacarescu, Cristian-Dragos Varganici, Daniela Ionita, Dragos Isac, Ana-Lavinia Vasiliu

**Affiliations:** 1 “Petru Poni” Institute of Macromolecular Chemistry, 700487 Iasi, Romania; amihaela@icmpp.ro (M.D.); dtimpu@icmpp.ro (D.T.); livius@icmpp.ro (L.S.); varganici.cristian@icmpp.ro (C.-D.V.); dgheorghiu@icmpp.ro (D.I.); isac.dragos@icmpp.ro (D.I.); vasiliu.lavinia@icmpp.ro (A.-L.V.); 2 Danish Polymer Centre, Department of Chemical and Biochemical Engineering, Technical University of Denmark, 2800 Kongens Lyngby, Denmark; lyyu@kt.dtu.dk

**Keywords:** interpenetrating polymer networks, silicone elastomers, wave energy harvesting, dielectric elastomer transducers

## Abstract

Interpenetrating polymer networks (IPNs) represent an interesting approach for tuning the properties of silicone elastomers due to the possible synergism that may occur between the networks. A new approach is presented, which consists of mixing two silicone-based networks with different crosslinking pathways; the first network being cured by condensation route and the second network by UV curing. The networks were mixed in different ratios and the resulted samples yield good mechanical properties (improved elongations, up to 720%, and Young’s modulus, 1 MPa), thermal properties (one glass transition temperature, ~−123 °C), good dielectric strength (~50 V/μm), and toughness (63 kJ/m^3^).

## 1. Introduction

Wave energy harvesting with dielectric elastomer transducers (DETs) is a relatively new technology with great application potential [1,2,3,4]. This technology is built around a variable capacitor, which is made of a dielectric elastomer with compliant electrodes on both sides.

Based on the crosslinking system used, the chemical backbone structure, and the type/amount of filler used, silicone elastomers can be soft or hard, biocompatible, or cytotoxic. Due to the nature of the siloxane bond, silicone elastomers operate over a wide temperature and humidity range, are highly flexible and elastic, and have high breakdown strengths and low Young’s modulus. Furthermore, using a specially engineered mold or a 3D printer, silicone elastomers can take nearly any shape. For these reasons, silicone elastomers can be used in a wide range of applications, including energy harvesting (e.g., wave energy harvesting [5,6], energy harvesting using human walking motions [7]), braille displays [8], robotics [9] (sensing skin), and they hold a significant share of the market for aeronautical applications as well (adhesives in spacecrafts and airplanes, components of wings, landing flaps, window gaskets, floor components) [10]. 

Silicone elastomers can be successfully used as dielectric elastomers in variable capacitors for wave energy harvesting due to the remarkable properties mentioned above, but their conversion efficiency is still limited due to a low dielectric permittivity and poor mechanical properties. 

Different strategies can be used to improve the dielectric permittivity and/or mechanical properties, such as adding polar groups to the polymer back-bone or in cross-linking nodes or using fillers with high dielectric permittivity.

The polar groups attached to the main polymer back-bone significantly reduce the mechanical properties and dielectric breakdown strengths of the cross-linked elastomers, even though they offer a high dielectric permittivity [11,12,13]. Generally, many fillers with high dielectric permittivity are not compatible with the siloxane matrix. A compatibility protocol must be followed to incorporate them into the siloxane matrix without causing defects at the filler/matrix interface. This compatibility protocol is not always successful and it adds another costly step to the preparation process. In most studies, the reinforcing effect of the fillers resulted in an increase in Young’s modulus and a significant decrease in dielectric strength, highlighting an imperfect compatibility.

Engineering elastomers with polymer networks that are interconnected after cross-linking, known as interpenetrated polymer networks (IPNs), is another strategy used to suppress the disadvantages of silicone elastomers. Thus far, many studies on IPNs have focused on actuation, achieving promising results. Full silicone IPNs as dielectric elastomers were first developed and studied by Brochu et al. [14]. They mixed a room temperature cross-linked network (RTV) with a high temperature cross-linked network (HTV) aiming to achieve a certain percent of preserved prestrained to increase the adhesion between dielectric layers. Materials exhibited over 20% actuation strains for more than 30,000 cycles while driving a load. Tugui et al. [15] followed Brochu’s work and used polar RTV networks obtaining promising materials for low voltage actuations with increased dielectric permittivity when trifluoro-propyl and 3-cyanopropyl groups were used. In another approach, Madsen et al. [16] replaced the RTV network by an ionic cross-linked network between a low molecular weight carboxydecyl terminated PDMS and an amino-functional PDMS in different wt % ratios. The obtained networks have higher dielectric permittivity due to the ionic part of the IPNs, relatively high breakdown strengths, and good actuation values based on figure of merit (FOM) calculations.

Our new approach consists in mixing two RTV networks in different rations with different cross-linking pathways: condensation (RTV-C) and UV cure (RTV-UV). The green full silicone IPNs obtained by this method present several advantages in comparison to previously presented strategies, such as requiring no complex chemical reactions to add polar groups or including several steps in the preparation procedure needed for filler incorporation that may lead to defects in the membrane or phase separations. Thus, a more scalable and cheaper method to obtain tuned silicone-based dielectric elastomers is presented that lead to superior energy harvesting properties as compared to commercial products.

## 2. Experimental

### 2.1. Materials

The following reagents were used as received: hydroxyl-terminated polydimethylsiloxane (FD 80, Mn = 70,000 g/mol), from was purchased from Wacker Chemie AG; Tetraethylortosilicate (TEOS, Mn = 208.33 g/mol, d = 0.933 g/mL, assay = 98%), dibutyltin dilaurate (DBTDL, Mn = 631.56 g/mol, d = 1.066 g/mL, assay = 95%), and 2,2-dimethyl-2-phenylacetophenone (DMPA, Mn = 256.30 g/mol, assay = 99%) were purchased from Sigma-Aldrich; two vinyl-terminated polydimethylsiloxane (DMSV31, Mn = 28,000 g/mol, d = 0.97 g/mL, assay > 95% and DMSV22, Mn = 9600 g/mol, d = 0.97 g/mL, assay > 95%) from Gelest; mercapto-functional silicone (GP-367, 5.4% SH, Mn = 3647 g/mol) from Gennese Polymer Corporation, Burton, MI 48529, USA.

### 2.2. Equipments and Methods

Electrical breakdown tests were performed on an in-house-built device based on international standards (IEC 60243-1 (1998) and IEC 60243-2 (2001)) and film thicknesses were measured through microscopy of cross-sectional cuts. The distance between the spherical electrodes was set accordingly with a micrometer stage and gauge. An indent of less than 5% of sample thickness was added, to ensure that the spheres were in contact with the sample. The polymer film was slid between the two spherical electrodes (diameter of 20 mm), and the breakdown was measured at the point of contact by applying a stepwise increasing voltage (50–100 V/step) at a rate of 0.5–1 steps/s. Each sample was subjected to 12 breakdown measurements, and an average of these values was given as the breakdown strength of the sample.

Dielectric relaxation spectroscopy (DRS) was performed on a Novocontrol Alpha-A high-performance frequency analyser (Novocontrol Technologies GmbH & Co, Montabaur, Germany) operating in the frequency range 10^−1^–10^6^ Hz at ambient temperature and at low electrical field (~1 V/mm). The dimeter of the electrode used was 20 mm.

The stress/strain curves were recorded on a Shimadzu AGS-J deformation apparatus at ambient temperature and at a rate of deformation of 200 mm/min with a load cell capable of measuring forces up to 1 kN and a sample film of 25 mm × 5 mm. For each point, five samples were tested and the average value was taken.

The small-angle X-ray scattering experiments (SAXS) were performed on a Nanostar U-Bruker system equipped with a Vantec 2000 detector (diameter of 200 mm) and an X-ray I μS microsource. The wavelength of the incident X-ray beam was λ = 1.54 Å (Cu Kα) and the beam was collimated by three pinholes. The scattered intensity I (q) was plotted as a function of the momentum transfer vector q = 4π sin θ/λ, where λ is the wavelength of the X-rays and θ is half the scattering angle. The sample-to-detector distance was 107 cm allowing measurements with q values between 0.008 Å^−1^ and 0.3 Å^−1^. The angular scale was calibrated by the scattering peaks of a silver behenate standard. The samples, in powder form were mounted on a dedicated holder using kapton foil and measured under vacuum at constant temperature, 25 °C for 10,000 s. The raw data was normalized for the transmission coefficient and the incoherent scattering due to background was subtracted using the SAXS–Bruker AXS software. The data analysis was done with the following software: *Bruker AXS* and “*Irena: tool suite for modeling and analysis of small-angle scattering*” [17,18].

Differential scanning calorimetry (DSC) measurements were conducted on a 200 F3 Maia DSC (Netzsch, Germany) calibrated with indium. A total 10 mg of sample was heated in aluminum crucibles with punched and sealed lids in nitrogen purge gas (flow rate 50 mL/min) and at a heating rate of 10 °C/min.

The computations were carried out with Gaussian G16 software [19]. Quantum mechanics (QM) method was selected for computations that included a description of the physical properties at the scale of atoms and subatomic particles (electrons) by density functional theory (DFT). DFT calculations have been reported to provide reliable results that balance the description of the optimized geometries with a good estimation of the energy gaps in π-conjugated oligomers and polymers [20]. As functional for computation, LC-wPBE [21] and 6–31 G (d,p) basis set was selected. The computational determination includes an optimization of a network node structure not on the entire polymer matrix. The network node structure included two compounds in computations: A–Figure S1 and B–Figure S2. The equilibrium molecular geometry was tested by Hessian matrix calculation and no imaginary frequency was obtained. This calculation indicated that optimized molecular geometry corresponds to a minimum of the potential energy. Additionally, the dipole moment and the partial atomic charges such as molecular electrostatic potential (MEP) surface, atomic polarizability tensor (APT), and the HOMO-LUMO (Highest Occupied Molecular Orbital-Lowest Unoccupied Molecular Orbital) energy gap were calculated with the DFT method. Based on these calculations, it could be estimated why a molecular system has a good permittivity (compound B) and another slightly lower (compound A).

Rheological characterization of the prepared films was performed with a TA Instruments ARES-G2 Rheometer set to 2% controlled strain mode, thus helping to stay within the linear viscoelastic regime. Measurements were performed with a parallel plate geometry of 8 mm at room temperature, with a normal force of 7 N and in the frequency range 100–0.01 Hz.

Atomic force microscopy (AFM) measurements for the topography of the samples were performed at room temperature and under ambient pressure, using a Scanning Probe Microscope SOLVER PRO-M AFM, NT-MDT (Russia). The images of the film surfaces were taken using the tapping mode with a high resolution no-contact silicon NSG10 cantilever. In all AFM measurements the scan range was 10 μm in the X-Y direction. Imagine acquisition and roughness parameters measurements were done with Nova 1.0.26.1443 software provided by NT-MDT. For a set of *n* numbers or values of a discrete distribution x_1_, x_2_, ……, x_*n*_, the root-mean-square (abbreviated “RMS”) is defined as:

$$RMN=\sqrt{\frac{x_{1}^{2}+x_{2}^{2}+\ldots +x_{n}^{2}}{n}};$$

where: *RMN* = root mean square; *n* = number of measurements; x_i_ = each value.

### 2.3. Sample Preparation

**Scheme 1:**
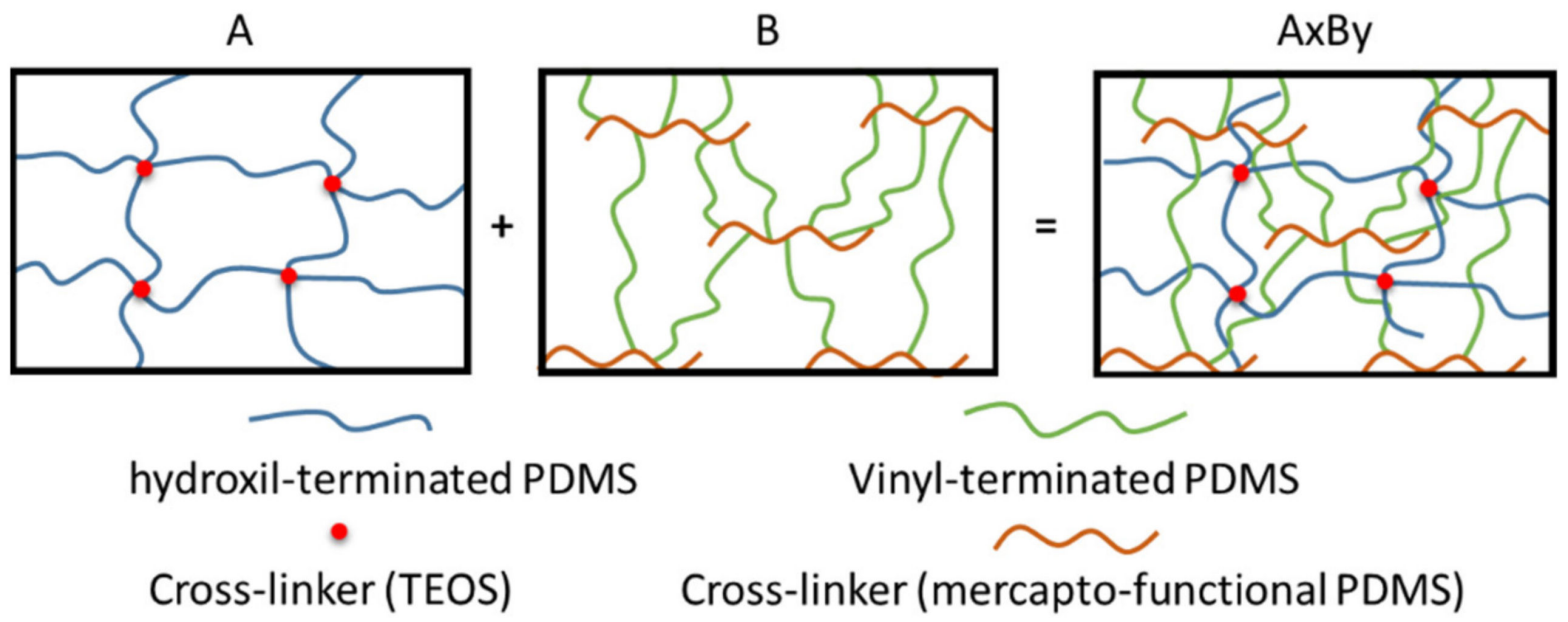
Schematic representation of the silicone-based IPNs formation.

The samples were obtained based on two siloxane-based polymer networks combined in different ratios and crosslinked by different pathways, as described in Table 1. Proper amounts, as described in Table S1, of hydroxyl-terminated polydimethylsiloxane (FD-80), which was used to obtain the first polymer network (A), and a vinyl-terminated polydimethylsiloxane (DMSV31 or DMSV22), used to obtain the second polymer network (B or C), were dissolved in a laboratory beaker for 15 min in toluene under rigorous stirring. After dissolving the polymers, TEOS, used as condensation curing crosslinker for network A, and a low molecular weight mercapto functional silicone (GP-367), used to cure network B or C by UV thiol-ene reaction, were added and mixed for 10 min. Previously dissolved DMPA, used as photo initiator in thiol-ene crosslinking, and DBTDL catalyst for condensation cure, were finally added, and stirring was maintained for another 2 min. The final mixture was poured on a Teflon petri dish and left for 15 min under UV radiation (A UV lamp G20T10 UVC 20W from Sankyo Denki, which emits ultraviolet radiations at a wavelength of 253.7 nm, with a UV output of 7.5 W was used in the experiments) to crosslink network B or C. After UV radiation, the samples were kept in laboratory condition for 12 h for the condensation cure to take place and successfully crosslink network A. All samples were easily peeled off the substrate and kept for another 4 weeks in laboratory conditions for ageing before further characterizations. The networks are schematically represented in Scheme 1.

## 3. Results and Discussions

**Figure 1:**
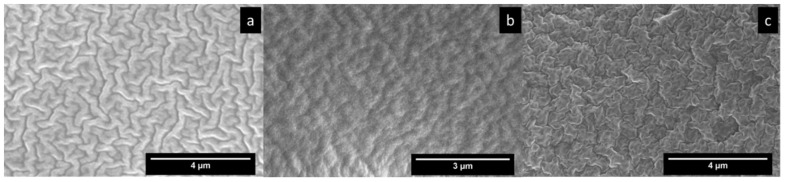
Cross-section morphology of samples A (**a**), B (**b**), and C (**c**); 4 μm scale.

Based on an elastic and stiff network, new silicone-based IPNs have been designed. The elastic network was made of hydroxyl-terminated PDMS with a high molecular weight, which was cross-linked with TEOS by condensation (A network). A low molecular weight vinyl-terminated PDMS network was cross-linked with a mercapto functional cross-linker via thiol-ene addition. Two low molecular weight vinyl-terminated PDMSs were selected to investigate the impact of the rigid network’s molecular weight (B and C network). Two sample series (AxBy and AxCy) were created by mixing the networks in varied weight percent proportions and a reference series (A–C). SEM was used to examine the cross-section morphology of the samples. A wrinkle-like morphology was noticed in sample A (Figure 1a), which is made of a polymer with a high molecular weight. A flake-like structure can be seen in sample C (Figure 1c) which is made of a low-molecular-weight polymer. Sample B, which is based on a polymer with an intermediate molecular weight compared to the other two (A and C), has a morphology that is a hybrid between the wrinkle-like and flake-like structures (Figure 1b).

**Figure 2:**
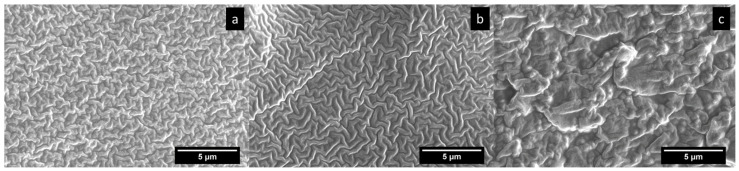
Cross-section morphology of samples A1B1 (**a**), A2B1 (**b**), and A1B2 (**c**); 5 μm scale.

The morphology of the AxBy (Figure 2) series is determined by the A network; the larger the network A’s weight ratio is, the more pronounced is the wrinkle-like structure. The flake-like structure is prevailing where more network B is present (Figure 2c). For instance, the 1:1 ratio (Figure 2a) offers a wrinkle-like morphology that is more dense and less pronounced than the 2:1 ratio (Figure 2b).

**Figure 3:**
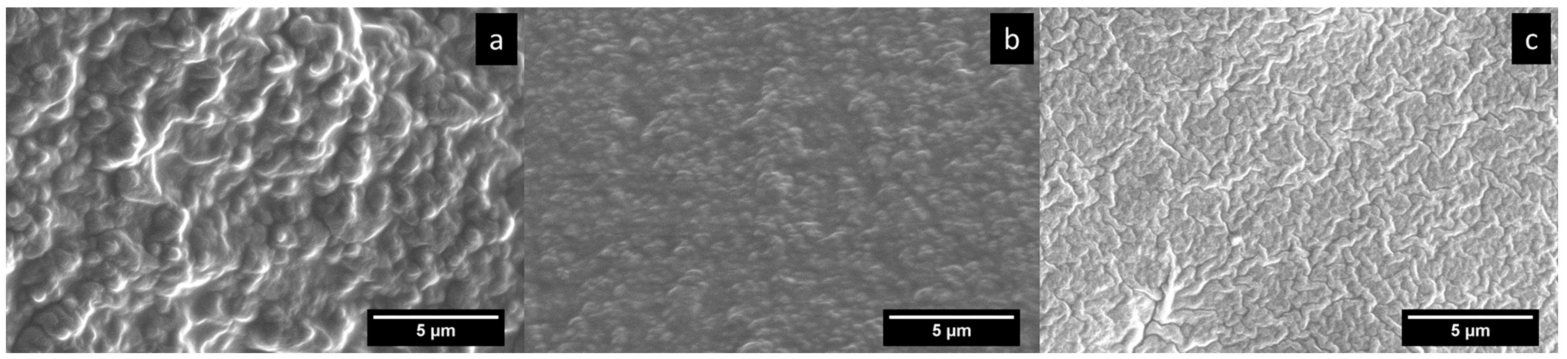
Cross-section morphology of samples A1C1 (**a**), A2C1 (**b**), and A1C2 (**c**); 5 μm scale.

In the case of the AxCy series (Figure 3), the same pattern can be observed. The network with the lower molecular weight, the rigid one, dictates the morphological structure of the samples, but in this case it changes dramatically. One can observe the appearance of a structure similar to spherical aggregates (Figure 3a) and a structure that is denser with smaller spherical aggregates when a higher ratio of the A network was used (Figure 3b).

The surface topography of polymer composite materials used in electronics is very important for the quality of contact with the metal surfaces of devices, in our case for the formation of capacitors and can influence the values for capacity, electrical permittivity, breakdown voltage, temperature factor, mechanical strength and adhesion to electrodes.

**Figure 4:**
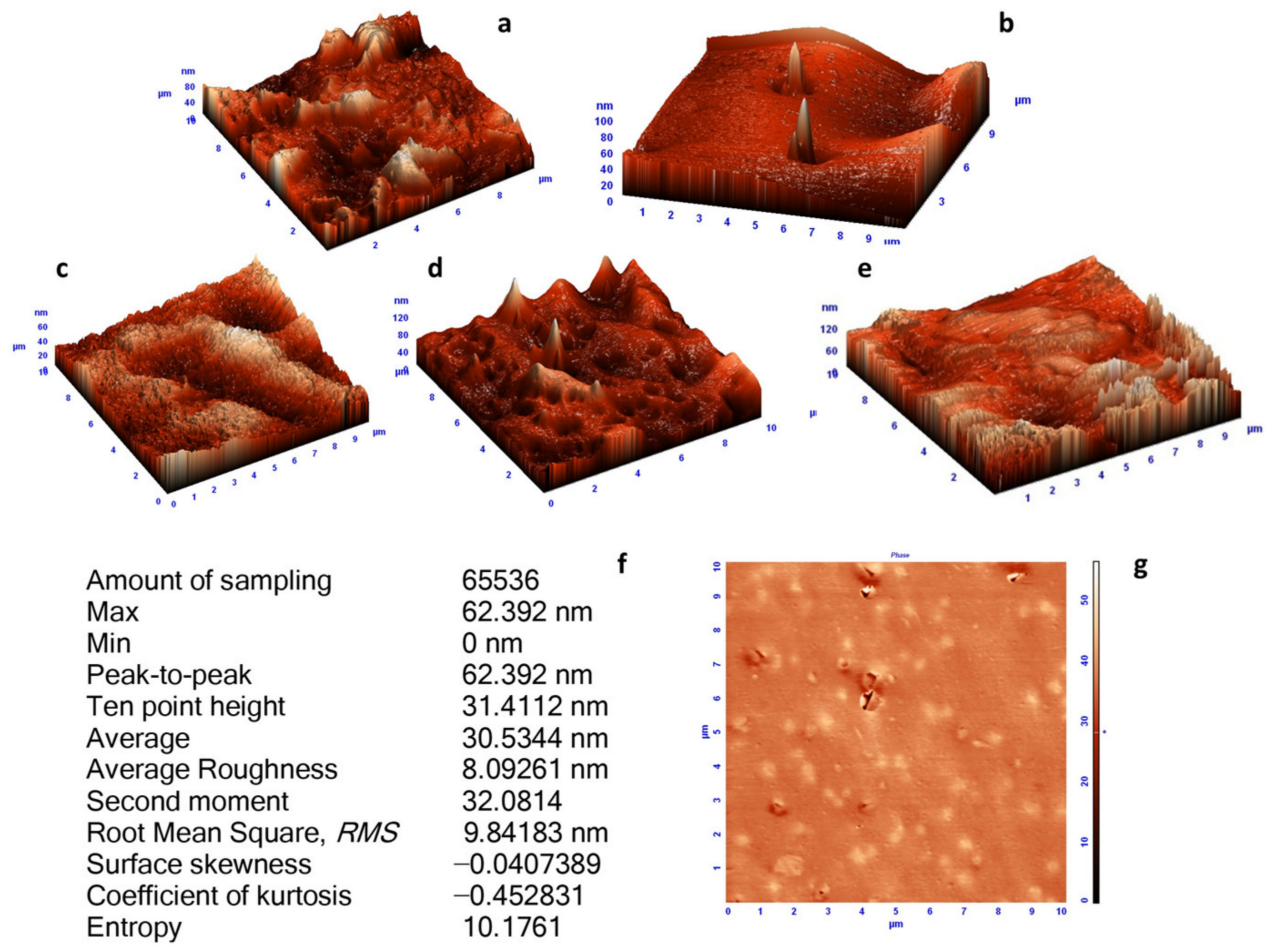
AFM scans of the reference samples (A–(**a**), C–(**b**)) and AxCy series (A1C1–(**c**), A1C2–(**d**), A2C1–(**e**)) and phase contrast for sample A1C1–(**g**), with statistical parameters of roughness-(**f**).

In this regard, the evaluation of surface roughness is one of the most important criteria. In many studies determination of the surface roughness was performed using a profilometer device. In the present study, AFM evaluation was used, which is a modern and precise method. The Figure 4 shows the 3D images and phase contrast for A and C precursors and for AxCy samples, for a 10 × 10 μm scan area.

For all investigated samples, RMS is below 0.05 microns, therefore, it is suitable for use in variable capacitor systems for wave energy harvesting with dielectric elastomer transducers. AFM investigations of the polymer network films revealed no considerable transformation of the surface’s morphology from precursors to polymer networks samples. The value of phase contrast is about 10° ÷ 20° (on surface); this indicates a good homogeneity and there are no phase separations. 

**Figure 5:**
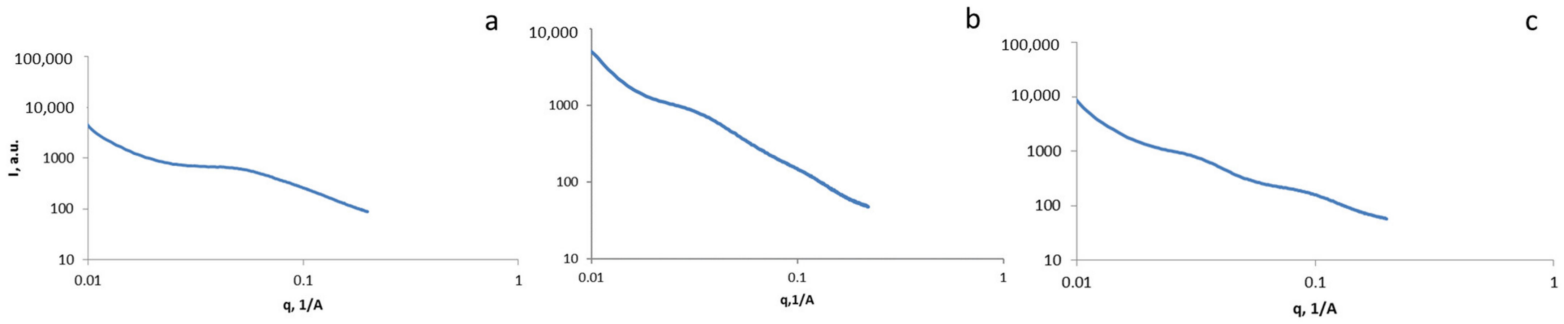
XR scattering curves in logarithmic scale for samples C (**a**), A2C1 (**b**), and A1C2 (**c**).

The spherical self-organized structures were analyzed by SAXS in comparison with C as a reference (pure PDMS network). The XR scattering curves in logarithmic scale as a result of SAXS characterization are showed in Figure 5. At low q values, the scattering curves obtained analyzing C and A1C2 samples (Figure 5a,c) have an upturn trend line behavior. This aspect is characteristic for samples where large aggregate structures without a clearly defined geometry are present. Therefore, samples C and A1C2 are made of densely cross-linked networks. The scattering curve obtained analyzing sample A2C1 (Figure 5b) has a Guinier region (I = constant) from which the following parameters can be estimated: Rg = 17 nm, Dmax = 2.58 × Rg = 43.86 nm, and corresponds to spherical self-assembling structures. Thus, the ratio with more A network (A2C1) leads to more space for network C to self-assemble in aggregates with uniform dimensions and shapes. In all cases, the scattering curves have a peak at low q values. This peak at q = 0.03 Å^−1^ corresponds to a Bragg distance of d = 20.9 nm in case of A1C2, and q = 0.05 Å^−1^ with d = 12.5 nm for sample C. In case of sample A2C1, this peak is at 0.035 Å^−1^ with a Bragg distance of d = 17.9 nm. It is well-known that the lower the q values are, the bigger the within organized structures are. The diffusion curves obtained analyzing the IPNs present a second peak at q = 0.096 Å^−1^ which correspond to a Bragg distance of d = 6.5 nm. This peak indicates a variation of electronic densities throughout significantly less distance as compared to sample C. 

**Figure 6:**
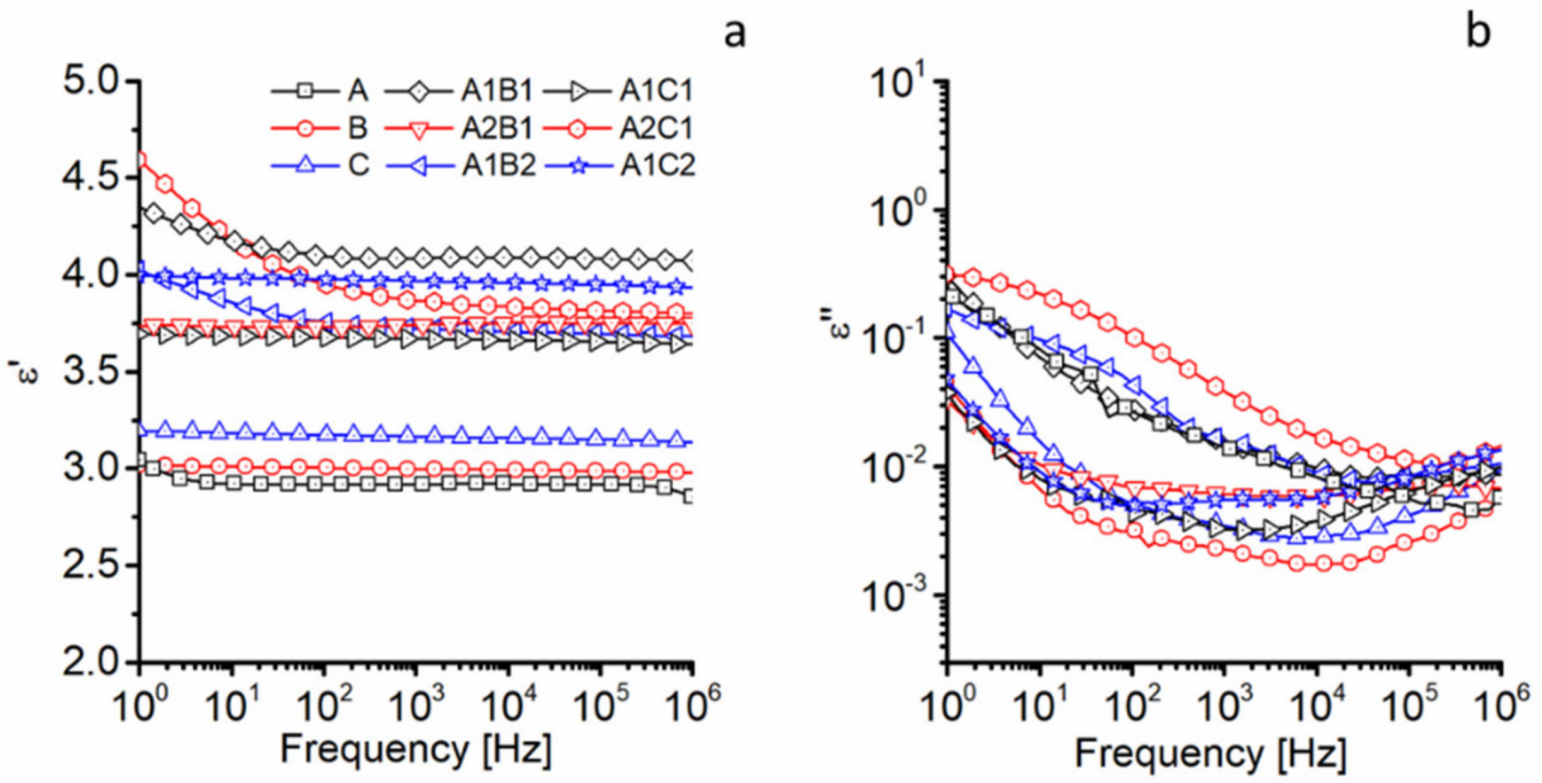
The dielectric spectra of the prepared samples; (**a**)–dielectric permittivity, (**b**)–dielectric loss.

Sample A possess a typical dielectric permittivity of 3 (Figure 6a), specific for pure silicone elastomers (PDMS). Samples B and C have a slight increase of the dielectric permittivity, due to the sulphur bridges formed after UV-induced thiol-ene crosslinking. The influence of the sulphur bridges on the dielectric properties were emphasised by a simplified computational study. The computational determination includes an optimization of a network node structure not on the entire polymer matrix (Figures S1 and S2). Theoretical results reveal that in case of the equilibrium molecular geometries, the values of dipole moments were 2.23 D for compound A and 4.63 D for molecular structure B (Figures S1 and S2). Introducing S atoms into the intramolecular configuration, the dipole moment value increases as an effect of polarity changing. Additionally, the presence of S atoms into the network nodes gives a supplementary negative charge as well as a large number of lone electron pairs, as compared to the network nodes A where only the oxygen atoms have the lone electron pairs. Moreover, the introduction of S atoms in the intramolecular fragments results in appearing of a new electronic configuration and the partial atomic charges are changed. As shown in Figures S3 and S4, compounds A and B contain atoms represented by positive and negative charges, thus forming polar bonds, especially in the compound B, making it more suitable for an increased permittivity. Furthermore, the synergy given by the combination of both networks provides an increased dielectric permittivity, around 4.2 (Figure 6a). The dielectric losses remain low, specific to silicone-based elastomers (Figure 6b).

**Figure 7:**
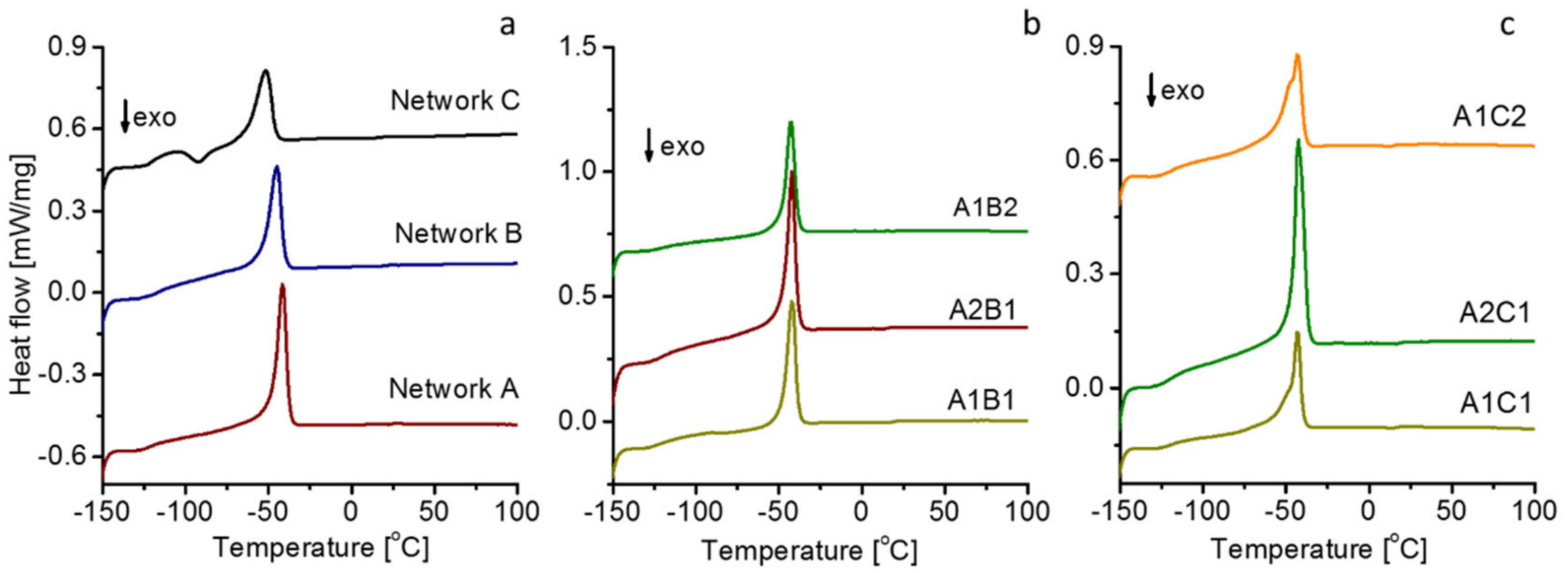
DSC second heating curves of the studied materials: (**a**)–Reference networks: A, B and C; (**b**)–AxBy series; (**c**)–AxCy series.

The glass transition temperature domain (T_g_) is an important parameter, since it determines polarization conditions and dipole concentration, orientation, and stability. The T_g_ further dictates thermal and mechanical properties of polymer-based materials. Hence, its determination by adequate techniques, such as DSC, is a crucial aspect in the characterization of multicomponent polymer-based materials [22,23]. It is also known that the presence of a single T_g_ domain is a sign of good phase miscibility between components, as it may be observed from Figure 7, Figure S5, and Table S2. The single T_g_ domain of the samples, around –120 °C, and the melting profile, around –43 °C, are typical to PDMS. During the obtaining of interpenetrating polymer networks, a synergism of the comprising components properties takes place by a forced phase compatibilization [24]. 

From Figure 7, a general decreasing tendency in the melting profile and the crystallinity degree may also be seen. An explanation resides in the disruption of the ordered conformation through hydrogen bonding, due to the formation of a new crystalline phase during the IPNs formation. This observation is in good agreement with literature data [24,25]. 

**Figure 8:**
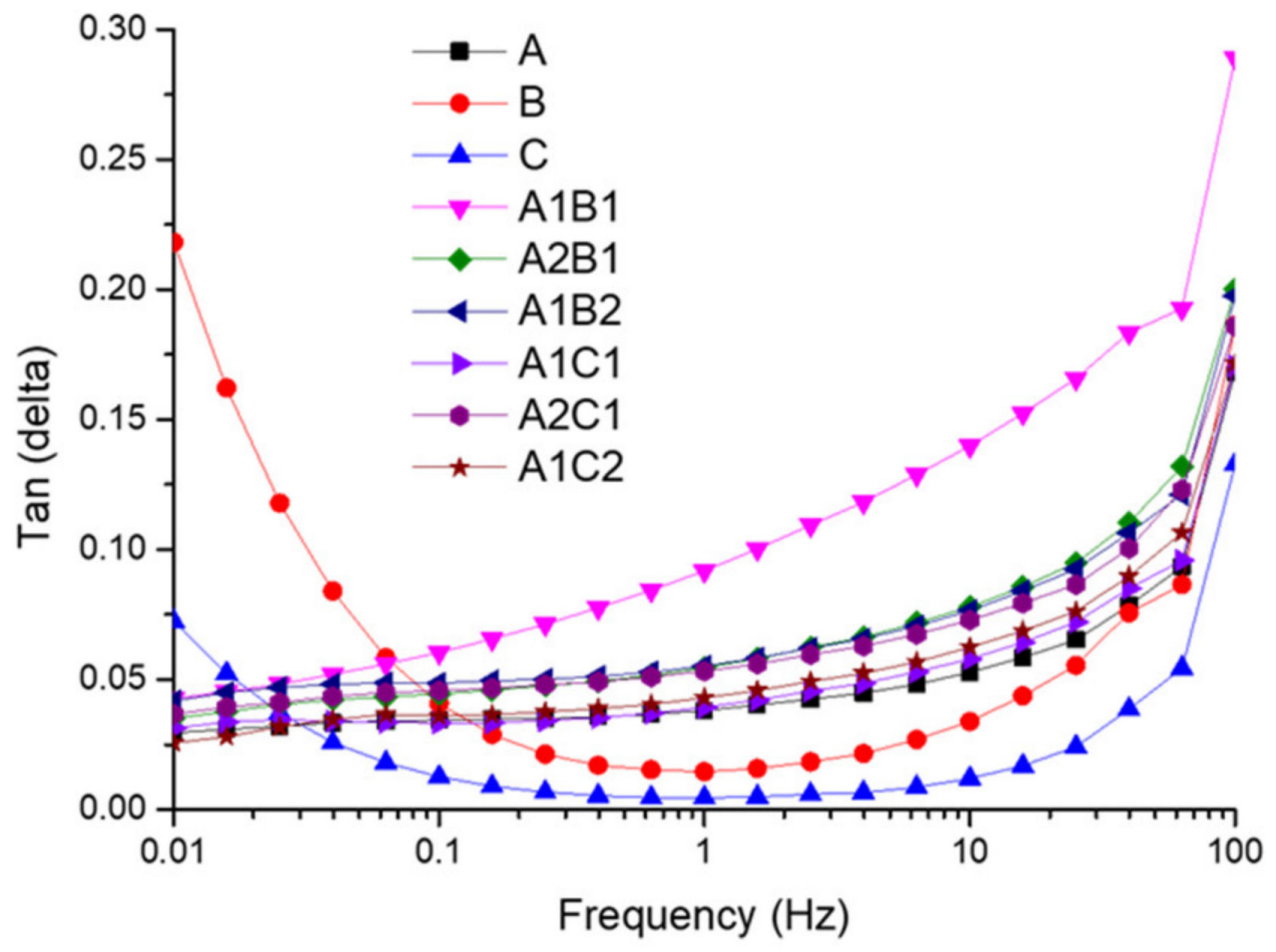
Low shear linear viscoelastic rheology of reference samples and the IPNs.

The loss tangent (tan δ = G”/G’) plot (Figure 8) provides some insight into the polymers’ molecular motion and damping behaviour. When compared to all other samples, the rigid network B has higher tan δ values. Sample B’s sharp increase in tan δ at lower frequencies (0.1–0.01 Hz) is due to the long relaxation times of the long dangling chains [26]. This is also visible for sample C, but due to the low molecular weight of the vinyl terminated PDMS, it is less pronounced. At these frequencies, however, no such relaxations were found in the IPNs. Furthermore, the prepared IPNs have significantly fewer dangling chains and lower viscous losses, as evidenced by the low tan δ values for the IPNs at lower frequencies.

**Figure 9:**
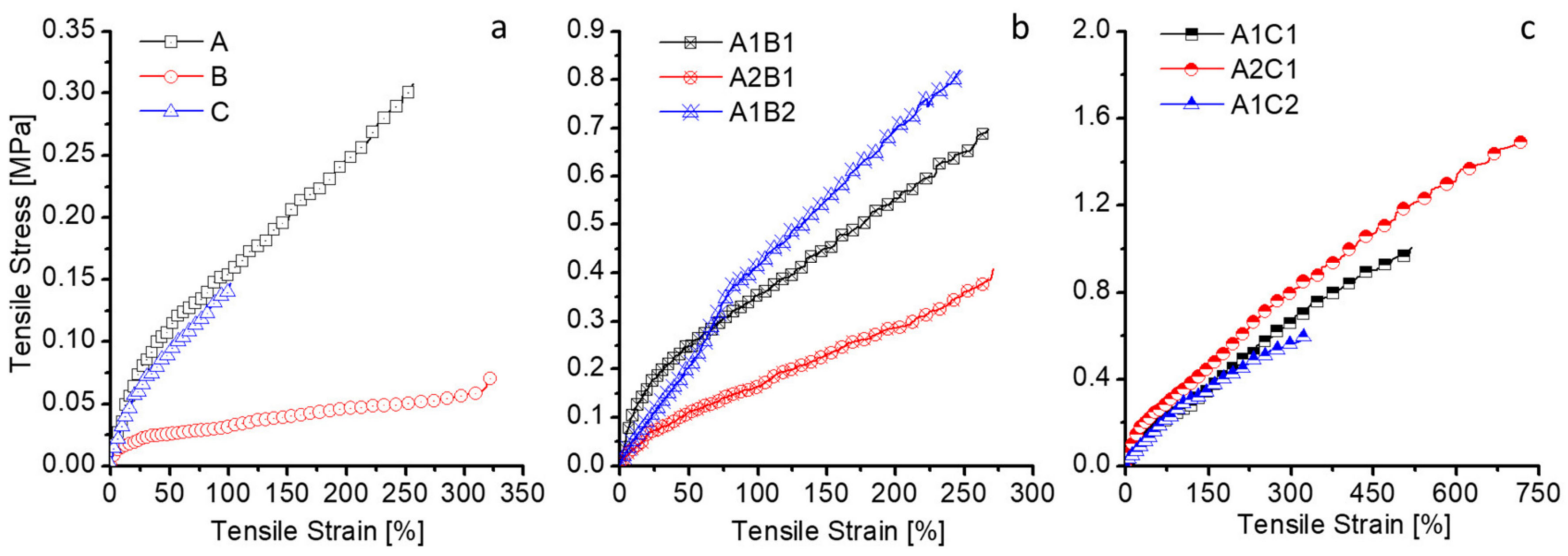
The mechanical properties (stress-strain curves) of the reference samples (**a**) and of the IPNs (**b**,**c**).

The elastomers obtained based on networks A, B, and C are soft with low tensile stresses and moderate elongations at break dictated by the molecular weights and the chosen cross-linking type. The long dangling chains emphasized by low shear linear viscoelastic rheology act as a plasticizer and give to sample B the lowest stress (0.1 MPa) and the highest elongation at break (330%), with a low value of Young’s modulus (calculated at 5% strain, 0.24 MPa). With a lower molecular weight of the vinyl terminated PDMS, the plasticizer effect is less pronounced, as in case of Sample C, having a decreased elongation at break (106%) and an increased Young’s modulus (0.47 MPa). The mechanical properties of the IPNs are drastically influenced by the presence of the rigid networks. The rigid network B acts as reinforcing agent, and the AxBy series have increased tensile stress and slightly decreased elongations at break (Figure 9, Table S4) compared to reference samples. Using a rigid network with a lower molecular weight (C), AxCy series have the highest tensile stress (1.74 MPa) and reach elongation at break more than twice compared to starting networks (720%). Moreover, the yield strength of both series (which denotes the elastic limit of the stress-strain curves) shifts to maximum elongation at break.

**Figure 10:**
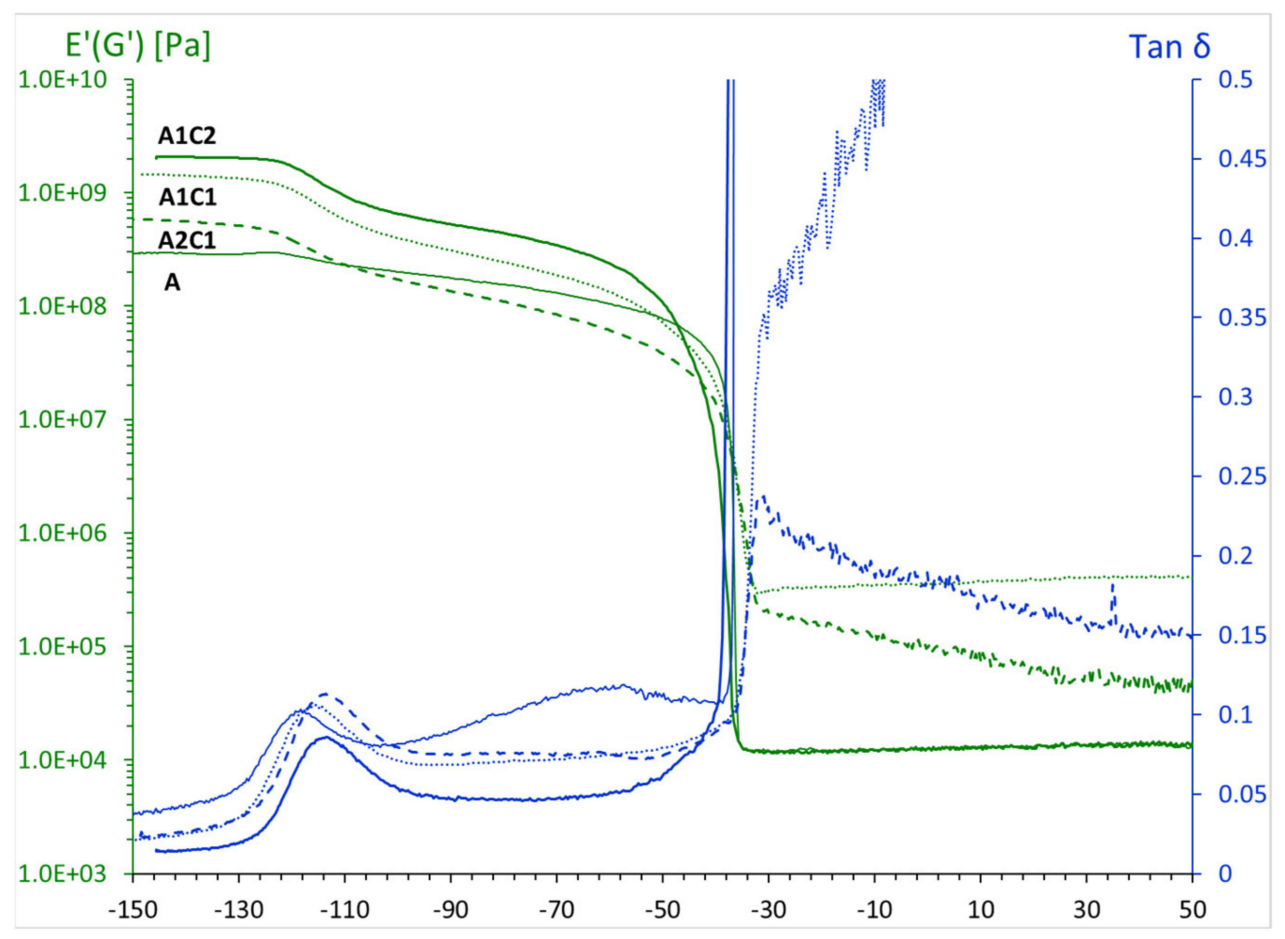
DMA characterization of reference sample A and AxCy series.

Single damping peaks are observed in the DMA scans (Figure 10), conforming a substantial interpenetration of the two networks. The corresponding damping peak temperatures, which are taken as glass transition temperatures (T_g_), are listed in Table S3. No important variations of melting temperatures can be observed, which are in good correlation with DSC (Figure 7). The shapes of tan δ peaks does not change too much, whereas the intensity and the position of the damping peak changes directly related to the A and C ratio. The presence of higher ratio of A enhances the mobility of the IPN, and the height of tan δ peak increases up to 0.11. The greater the C content is, the more rigid is the IPN structure. The lowest damping peak intensity (0.08) observed for the A1C2 suggests that the C network brings a strong restraining effect on the polymer network segmental mobility. This restraint is in accordance with the increasing trend of the storage modulus.

**Figure 11:**
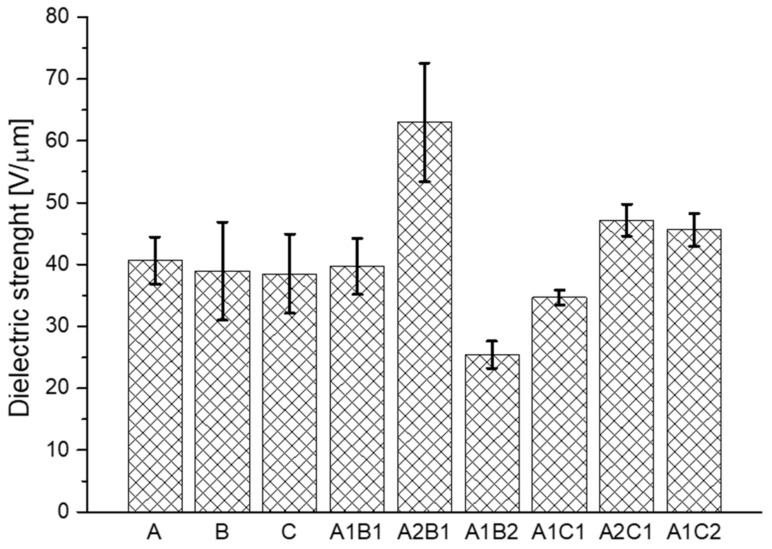
Dielectric strength of the prepared samples.

The breakdown strength of ratio 1:1 IPNs (A1B1 and A1C1) is similar to reference samples (A, B, C), Figure 11. While in ratio 2:1 (A2B1 and A2C1), the breakdown strength is higher than the reference ones. This might be due to the to the presence of free un-crosslinked chains that diminished in the IPNs in the 2:1 ratio, resulting in the suppression of electromechanical instability and consequently enhancing the breakdown strength. However, when the content of UV-crosslinked PDMS is increased, the Ebd of A1B2 is dramatically deteriorated due to the morphology irregularities (Figure 2c) causing more electromechanical instability or voids in the network. Nevertheless, the morphology between A2C1 and A1C2 are similar (Figure 3b,c), which is more homogenous than A1C1 (Figure 3a), therefore both exhibiting higher breakdown strength than reference monomodal (A, C) and A1C1 samples.

Toughness (*UTT*, Equation (1)) refers to how much energy a material can absorb per unit volume before rupturing. Having improved elongation at break, sample A2C1 has the highest value, exceeding the commercial silicone elastomer Elastosil 3060.

(1) $$UTT=\int_{0}^{S_{\max}}T_{\mathrm{nm}}dS$$

(2) $$\frac{\Delta W}{V}=0.5\varepsilon^{\prime }\varepsilon_{0}U^{2}\left(1-\frac{1}{{\left(S_{\max}+1\right)}^{2}}\right)$$

where *UTT*—Toughness [kJ/m^3^]; *T*_nm_—Tensile stress [MPa]; *S*_max_—elongation at break [%]; Δ*W*/*V*—energy output [mJ/cm^3^]; *ε*′—dielectric permittivity; *ε*_0_—dielectric permittivity of free space [F/m]; *U*—applied electric field [25 V/μm].

**Figure 12:**
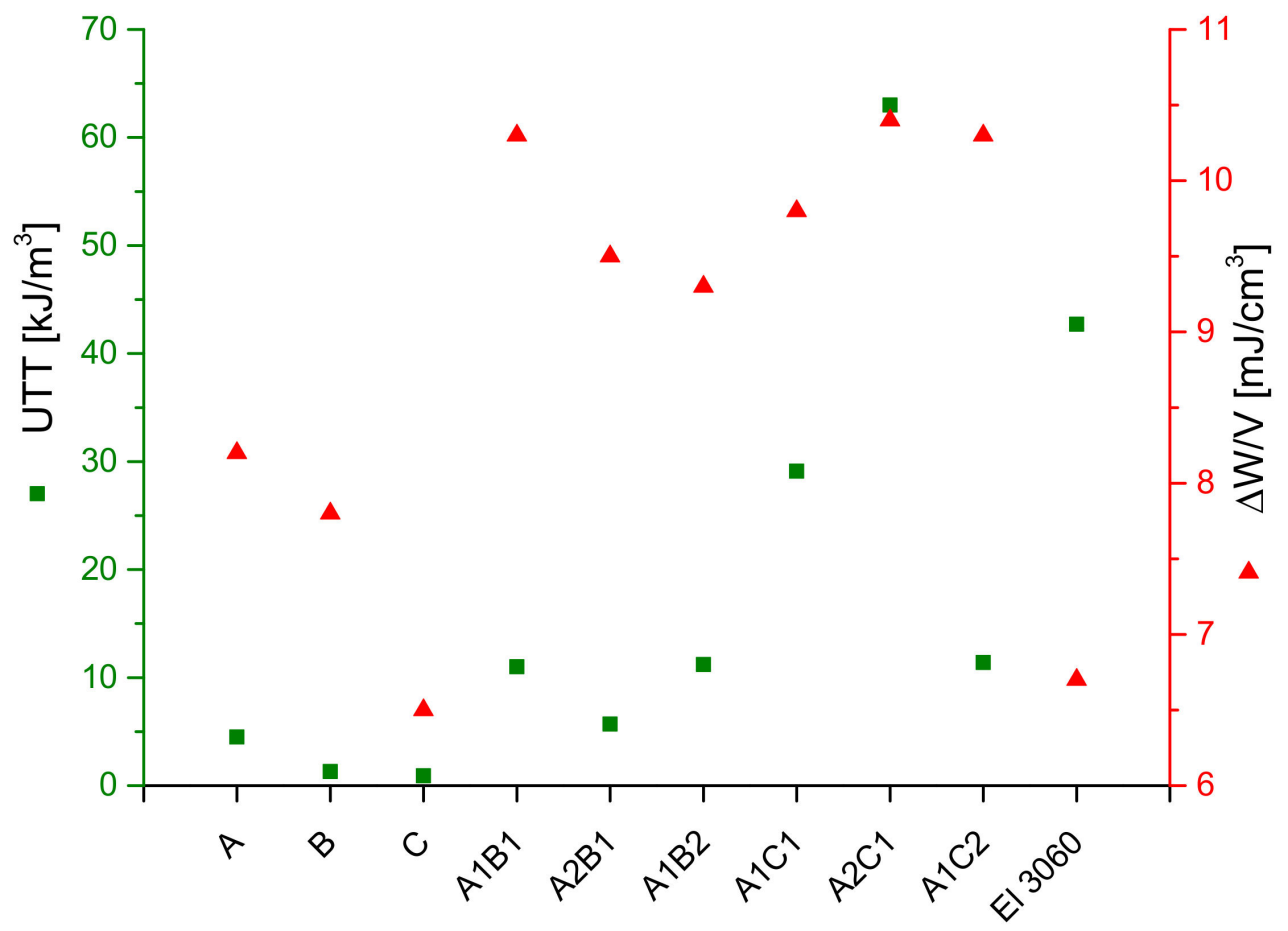
Toughness and energy output of the prepared samples.

The energy that can be harvested from one cycle of elongation-relaxing can be estimated from Equation (2). It can be noticed that the estimated energy output (Δ*W*/*V*) is in good correlation with toughness and the highest values were obtained for sample A2C1 with the best mechanical properties and toughness (Figure 12).

## 4. Conclusions

New silicone-based IPNs were developed based on an elastic network and a rigid one. The elastic network was a high molecular weight hydroxyl-terminated PDMS cross-linked via condensation route with TEOS (A network). The rigid network was a low molecular weight vinyl-terminated PDMS crosslinked via thiol-ene addition with a mercapto functional crosslinker. To study the influence of the molecular weight of the rigid network, two low molecular weight vinyl-terminated PDMSs were chosen (DMSV31 with Mn = 28,000 g/mol and DMSV22, with Mn = 9600 g/mol). The networks were mixed in different wt % rations to give two sample series (AxBy and AxCy) and the reference series (A–C). The wt % ratio and the molecular weight of the vinyl-terminated PDMS had great influence. The cross-section morphology emphasized wrinkle-like and flake-like structures in correlation with the wt % ratio used. The wrinkle-like structure is more pronounced when network A’s weight ratio is higher, and vice versa, the flake-like structure is prevailing where more network B was used. In case of network C, the morphology changes and spherical aggregates formed, which are denser and smaller when a higher ratio of the A network was used. The large aggregate wrinkle-like structures without a clearly defined geometry were demonstrated by SAXS and the spherical aggregates proved to be around 43.86 nm in size. The AFM scans showed no phase separation, which is in good correlation with a single glass transition temperature emphasized by DMA and DSC. Additionally, tan δ values reveal that the sample A2C1 (ratio 2:1 between the elastic and the rigid network) is the most suitable combination, which is in good correlation with the mechanical properties. Sample A2C1 gives the highest elongation at break (720%), achieving high toughness (63 kJ/m^3^) and energy output (10.45 mJ/cm^3^).

## Figures and Tables

**Table 1:** The wt % ratios used to obtain the reference samples and the IPNs (AxBy or AxCy).

Network	Sample								
	A	B	C	A1B1	A2B1	A1B2	A1C1	A2C1	A1C2
A *	1	0	0	1	2	1	1	2	1
B **	0	1	0	1	1	2	0	0	0
C **	0	0	1	0	0	0	1	1	2

*—condensation cross-linking; **—UV cross-linking; x and y stands for network rations.

## Data Availability

Not applicable.

## Supplementary Materials

The following supporting information can be downloaded at: https://www.mdpi.com/article/10.3390/polym14010211/s1, Figure S1: Representation of the equilibrium geometry and dipole moment orientation for structure A into the ground state. Theoretical calculations performed with the LC-wPBE/6-31G(d,p) method; Figure S2: Representation of the equilibrium geometry and dipole moment orientation in the case of the structure B (or C) into the ground state. Theoretical calculations performed with the LC-WPBE/6-31G(d,p) method; Figure S3: Electrostatic potential rendered as a mapped surface in the vicinity of molecule A (computation done at LC-wPBE/6-31G(d,p) level of theory); Figure S4: Electrostatic potential rendered as a mapped surface in the vicinity of molecule A (computation done at LC-wPBE/6-31G(d,p) level of theory); Table S1: The amounts used for obtaining the IPNs and the reference samples; Table S2: Thermal characteristics extracted from DSC data; Figure S5: Inset of DSC data emphasizing the glass transition temperature; Table S3: DMA data of reference sample A and AxCy series; Table S4: Mechanical and dielectric data of prepared samples in comparison with representative IPNs from literature.
