# Supplementary material for: Binary Silicone Elastomeric Systems with Stepwise Crosslinking as a Tool for Tuning Electromechanical Behavior
Source: Polymers (Basel). 2022 Jan 5;14(1):211. doi: 10.3390/polym14010211 (PMC8747420; doi:10.3390/polym14010211)
Supplement: Supplementary file 1 [file polymers-14-00211-s001.zip › polymers-1497684-supplementary.pdf]

polymers

MDPI

*Supplementary Materials*

# Binary Silicone Elastomeric Systems with Stepwise Crosslinking as a Tool for Tuning Electromechanical Behavior

**Adrian Bele [1,*], Liyun Yu [2], Dascalu Mihaela [1], Daniel Timpu [1], Liviu Sacarescu [1], Cristian-Dragos Varganici [1], Daniela Ionita [1], Dragos Isac [1] and Lavinia Matricala [1]**

1 "Petru Poni" Institute of Macromolecular Chemistry, Iasi, 700487, Romania; amihaela@icmpp.ro (D.M.); dtimpu@icmpp.ro (D.T.); livius@icmpp.ro (L.S.); varganici.cristian@icmpp.ro (C.-D.V.); dgheorghiu@icmpp.ro (D.I.); isac.dragos@icmpp.ro (D.I.); vasiliu.lavinia@icmpp.ro (L.M.)

2 Danish Polymer Centre, Department of Chemical and Biochemical Engineering, Technical University of Denmark, Kongens Lyngby, 2800, Denmark; lyyu@kt.dtu.dk

* Correspondence: bele.adrian@icmpp.ro

**Citation:** Bele, A.; Yu, L.; Mihaela, D.; Timpu, D.; Sacarescu, L.; Varganici, C.-D.; Ionita, D.; Isac, D.; Matricala, L. Binary Silicone Elastomeric Systems with Stepwise Crosslinking as a Tool for Tuning Electromechanical Behavior. *Polymers* **2022**, *14*, 200. https://doi.org/10.3390/polym14010200

Academic Editor: Marcin Masłowski

Received: 22 November 2021
Accepted: 29 December 2021
Published: 4 January 2022

**Publisher's Note:** MDPI stays neutral with regard to jurisdictional claims in published maps and institutional affiliations.

**Copyright:** © 2022 by the authors. Licensee MDPI, Basel, Switzerland. This article is an open access article distributed under the terms and conditions of the Creative Commons Attribution (CC BY) license (http://creativecommons.org/licenses/by/4.0/).

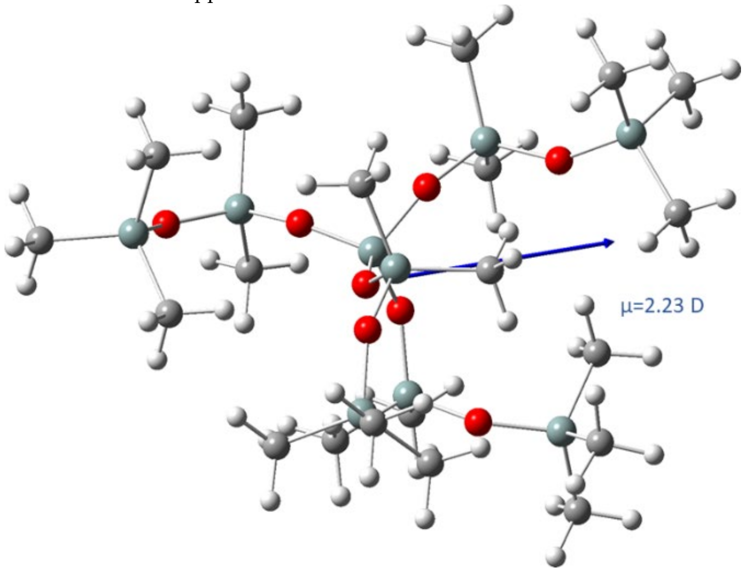


**Figure S1.** Representation of the equilibrium geometry and dipole moment orientation for structure A into the ground state. Theoretical calculations performed with the LC-wPBE/6-31G(d,p) method.

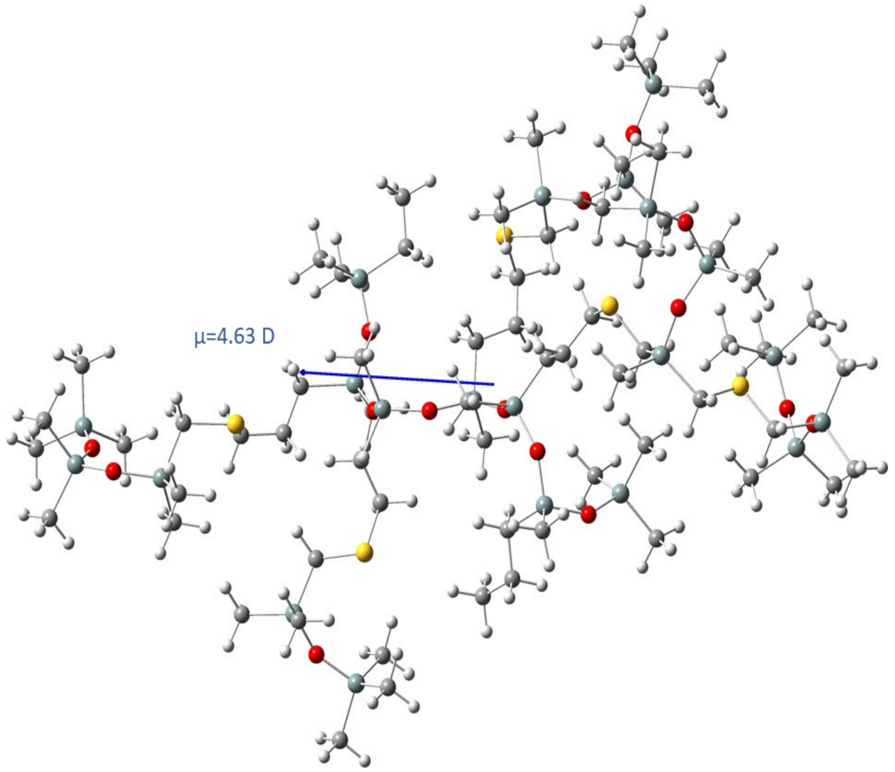


**Figure S2.** Representation of the equilibrium geometry and dipole moment orientation in the case of the structure B (or C) into the ground state. Theoretical calculations performed with the LC-WPBE/6-31G(d,p) method.

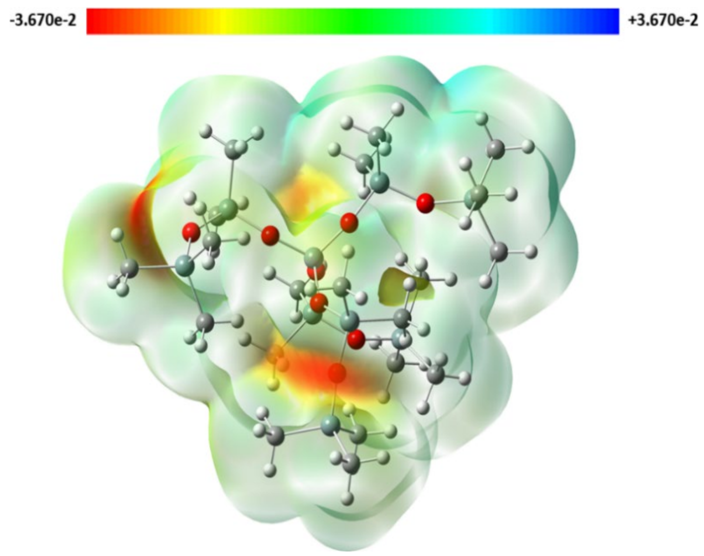


**Figure S3.** Electrostatic potential rendered as a mapped surface in the vicinity of molecule A (computation done at LC-wPBE/6-31G(d,p) level of theory).

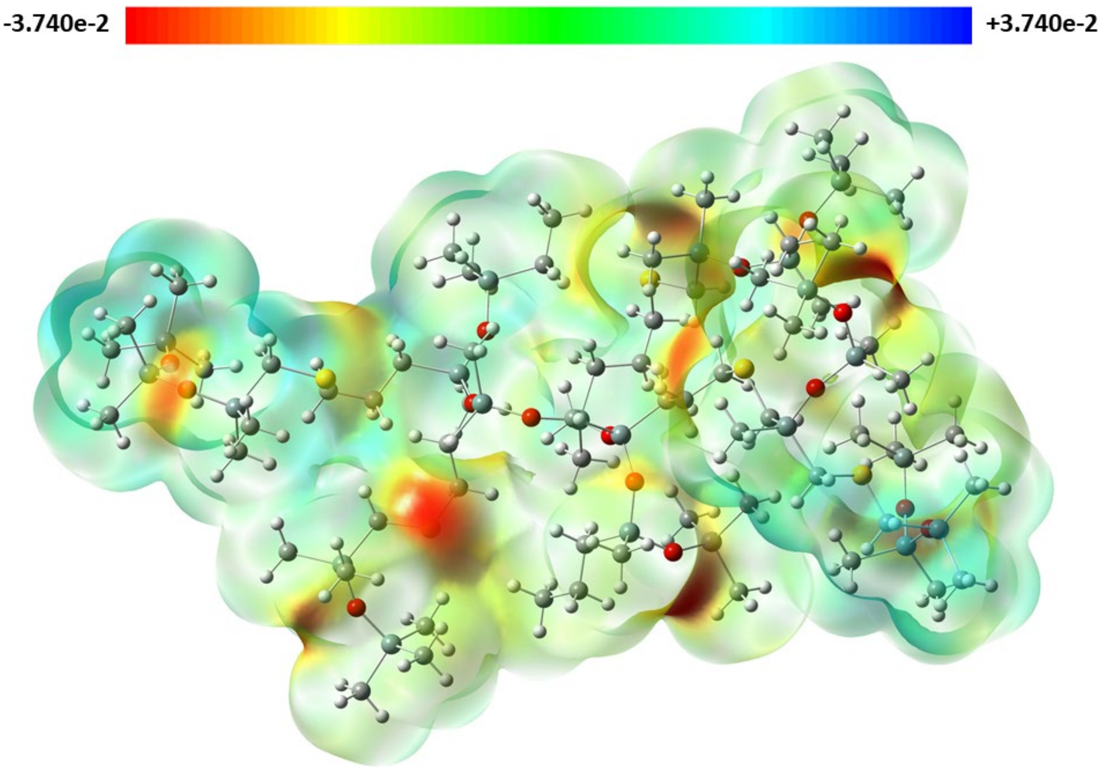


**Figure S4.** Electrostatic potential rendered as a mapped surface in the vicinity of molecule A (computation done at LC-wPBE/6-31G(d,p) level of theory).

Table S1. The amounts used for obtaining the IPNs and the reference samples

| Reagent/Sample | A | | B | | C | | A1B1 | | A2B1 | | A1B2 | | A1C1 | | A2C1 | | A1C2 | |
|---|---|---|---|---|---|---|---|---|---|---|---|---|---|---|---|---|---|---|
| | g | mmol | g | mmol | g | mmol | g | mmol | g | mmol | g | mmol | g | mmol | g | mmol | g | mmol |
| FD 80 | 2 | 0.028 | | | | | 2 | 0.028 | 2 | 0.028 | 1 | 0.014 | 2 | 0.028 | 2 | 0.028 | 1 | 0.014 |
| DMSV31 | | | 2 | 0.071 | | | 2 | 0.071 | 1 | 0.014 | 2 | 0.071 | | | | | | |
| DMSV22 | | | | | 2 | 0.21 | | | | | | | 2 | 0.21 | 1 | 0.106 | 2 | 0.21 |
| TEOS | 0.93 | 4.48 | | | | | 0.93 | 4.48 | 0.93 | 4.48 | 0.466 | 2.236 | 0.93 | 4.48 | 0.93 | 4.48 | 0.466 | 2.236 |
| DBTDL | 0.01 | 0.016 | | | | | 0.01 | 0.016 | 0.01 | 0.016 | 0.005 | 0.008 | 0.01 | 0.016 | 0.01 | 0.016 | 0.005 | 0.008 |
| GP-367 | | | 0.176 | 0.048 | 0.258 | 0.071 | 0.176 | 0.048 | 0.088 | 0.024 | 0.176 | 0.048 | 0.258 | 0.071 | 0.129 | 0.035 | 0.258 | 0.071 |
| DMPA | | | 0.108 | 0.42 | 0.113 | 0.44 | 0.108 | 0.42 | 0.054 | 0.21 | 0.108 | 0.42 | 0.113 | 0.44 | 0.056 | 0.218 | 0.113 | 0.44 |

Table S2. Thermal characteristics extracted from DSC data

| Sample | $T_{g1}$ (°C) | $T_{g2}$ (°C) | $T_{m1}$ (°C) | $\Delta H_{m1}$ (J/g) | $T_{m2}$ (°C) | $\Delta H_{m2}$ (J/g) | $T_{cr}$ (°C) | $\Delta H_{cr}$ (J/g) | $C_p$ (J/g °C) | $\chi_{PDMS/IPN}$ (%) |
|---|---|---|---|---|---|---|---|---|---|---|
| PDMS | − 122 | − 120 | − 42 | 23.46 | − 42 | 24.55 | − 68 | − 24.34 | 0.067 | 0.40 |
| A | − 123 | − 123 | − 42 | 24.73 | − 42 | 24.67 | − 70 | − 24.24 | 0.074 | 0.40 |
| B | − 119 | − 119 | − 45 | 21.52 | − 45 | 21.15 | − 81 | − 22.27 | 0.111 | 0.34 |
| C | − 121 | − 121 | − 52 | 16.25 | − 52 | 16.81 | − 94 | − 10.42 | 0.115 | 0.27 |
| A1B1 | − 121 | − 121 | − 43 | 19.36 | − 42 | 20.34 | − 70 | − 21.17 | 0.151 | 0.33 |
| A2B1 | − 122 | − 122 | − 43 | 24.55 | − 42 | 26.86 | − 70 | − 24.86 | 0.108 | 0.40 |
| A1B2 | − 120 | − 121 | − 43 | 17.35 | − 43 | 17.98 | − 73 | − 17.7 | 0.079 | 0.29 |
| A1C1 | − 119 | − 119 | − 43 | 12.53 | − 43 | 12.55 | − 70 | − 14.12 | 0.083 | 0.20 |
| A2C1 | − 120 | − 120 | − 43 | 24.29 | − 43 | 24.9 | − 70 | − 22.7 | 0.086 | 0.40 |
| A1C2 | − 117 | − 117 | − 43 | 17.84 | − 43 | 17.78 | − 71 | − 17.5 | 0.096 | 0.29 |

$T_{g1}$ – glass transition temperature corresponding to the first heating run; $T_{g2}$ – glass transition temperature corresponding to the second heating run; $T_{m1}$ – melting temperature corresponding to the first heating run; $T_{m2}$ – melting temperature corresponding to the second heating run; $\Delta H_{m1}$ – enthalpy of the melting profile corresponding to the first heating run; $\Delta H_{m2}$ – enthalpy of the melting profile corresponding to the second heating run; $T_{cc1}$ – cold crystallization temperature corresponding to the first heating run; $T_{cc2}$ – cold crystallization temperature corresponding to the second heating run; $\Delta H_{cc1}$ – enthalpy of the cold crystallization profile corresponding to the first heating run; $\Delta H_{cc2}$ – enthalpy of the cold crystallization profile corresponding to the second heating run; $\Delta H_{cr}$ – enthalpy of the crystallization profile; $C_p$ – heat capacity; $\chi_{PDMS/IPN}$ – degree of PDMS crystallinity in the networks.

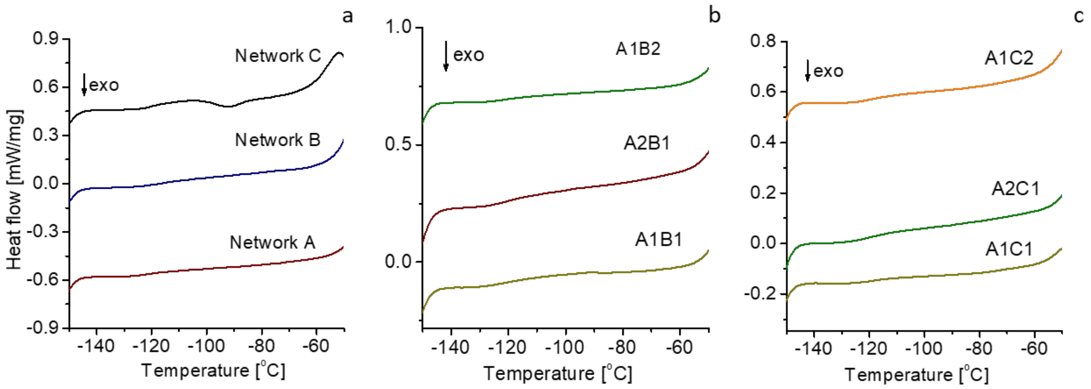


**Figure S5.** Inset of DSC data emphasizing the glass transition temperature.

Table S3. DMA data of reference sample A and AxCy series

| Sample | E′ (Pa) | $T_g$ | | $T_m$ (°C) |
|---|---|---|---|---|
| | | tan δ | T(°C) | |
| A | 2.94x10$^{8}$ | 0.101 | -118.08 | -36.13 |
| A2C1 | 5.75x10$^{8}$ | 0.112 | -112.9 | -35.5 |
| A1C1 | 1.44 x10$^{9}$ | 0.105 | -115.1 | -36.2 |
| A1C2 | 2.06x10$^{9}$ | 0.085 | -113.93 | -38.4 |

**Table S4.** Mechanical and dielectric data of prepared samples in comparison with representative IPNs from literature.

| **Sample** | **Sm (%)** | **Tnm (MPa)** | **Y (MPa)** | **ε'** | **Ebd (V/μm)** | **UTT, (kJ/m³)** | **ΔW/V (mJ/cm³)** |
|---|---|---|---|---|---|---|---|
| A | 256 | 0.31 | 0.42 | 3.25 | 41 | 4.50 | 8.23 |
| B | 325 | 0.07 | 0.22 | 3.00 | 39 | 1.30 | 7.79 |
| C | 100 | 0.15 | 0.41 | 3.17 | 39 | 0.90 | 6.54 |
| A1B1 | 267 | 0.70 | 1.27 | 4.08 | 40 | 11.00 | 10.39 |
| A2B1 | 270 | 0.41 | 0.42 | 3.72 | 63 | 5.70 | 9.48 |
| A1B2 | 247 | 0.81 | 0.61 | 3.71 | 25 | 11.20 | 9.36 |
| A1C1 | 520 | 1.00 | 0.60 | 3.66 | 35 | 29.10 | 9.80 |
| A2C1 | 720 | 1.50 | 1.00 | 3.86 | 47 | 63.00 | 10.46 |
| A1C2 | 320 | 0.60 | 0.45 | 3.97 | 46 | 11.40 | 10.30 |
| Elastosil 3060 | 310 | 3.15 | 1.24 | 2.58 | 90 | 42.70 | 6.67 |
| ref 14 (sample 15wt%) | 600 | 3.10 | -* | -* | 45 | -** | -* |
| ref 15 (sample IPN-P1) | 484 | 0.30 | 0.27 | 2.92 | 60 | -** | 9.00 |
| ref 15 (sample IPN-F2) | 512 | 0.35 | 0.11 | 3.01 | 12 | -** | 1.90*** |
| ref 15 (sample IPN-CN1) | 486 | 0.27 | 0.03 | 3.62 | 15 | -** | 3.60*** |
| ref 15 (sample IPN-F2) | 692 | 0.32 | 0.13 | 2.93 | 29 | -** | 8.10 |
| ref 16 (sample Si_A_IN10) | 560 | 3.30 | 0.26 | 3.30 | 55 | -** | 8.90 |
| ref 16 (sample Si_B_IN10) | 670 | 2.70 | 0.22 | 3.80 | 53 | -** | 10.1 |

Sm – Tensile strain (values given at break); Tnm – Tensile stress (values given at break); Y – Young's modulus (calculated at 5 % Sm); ε' – Dielectric permittivity (values given at $10^3$ Hz); UTT – Ultimate tensile toughness; ΔW/V - Energy output at 25 V/μm; * - values not available; ** - no values calculated due to the fact that raw mechanical data is needed in order to make calculations; *** - calculated at maximum Ebd provided by the authors.
